# Facile Synthesis and Characterization of Ibuprofen-mesoporous Hydroxyapatite Nanohybrid as a Sustained Drug Delivery System

**DOI:** 10.22037/ijpr.2019.1100769

**Published:** 2019

**Authors:** Zahra Namazi, Tahereh Sadat Jafarzadeh Kashi, Mohammad Erfan, Farhood Najafi, Leila Bakhtiari, Seyed Rohola Ghodsi, Hassan Farhadnejad

**Affiliations:** a *Department of Dental Biomaterials, School of Dentistry, Tehran University of Medical Sciences, Tehran, Iran. *; b *Iranian Tissue Bank and Research Center, Imam Khomeini Medical Complex Hospital, Tehran University of Medical Sciences, Tehran, Iran.*; c *Department of Pharmaceutics, School of Pharmacy, Shahid Beheshti University of Medical Sciences, Tehran, Iran.*; d *Department of Resin and Additives, Institute for Color Sciences and Technology, Tehran, Iran. *; e *SHEZAN Research and Innovation Center, Pardis Technology Park, Tehran, Iran.*; f *DanaWell Medical Equipment Company, Dental Equipment and Bio-material Technology Incubation Center, Tehran University of Medical Sciences, Tehran, Iran. *; g *Student Research Committee, Department of Pharmaceutics and Pharmaceutical Nanotechnology, School of Pharmacy, Shahid Beheshti University of Medical Sciences, Tehran, Iran.*

**Keywords:** Mesoporous, Nanoparticle, Hydroxyapatite, Ibuprofen, Drug Delivery

## Abstract

The present study deals with the fabrication of ibuprofen-mesoporous hydroxyapatite (IBU-MHA) particles via the incorporation of ibuprofen (IBU)—as a nonsteroidal anti-inflammatory drug—into mesoporous hydroxyapatite nanoparticles (MHANPs) using an impregnation process, as a novel drug delivery device. MHANPs were synthesized by a self-assembly process using cetyltrimethylammonium bromide (CTAB) as a cationic surfactant and 1-dodecanethiol as a pore expander under basic condition. The focus of the present study was to optimize the incorporation of IBU molecules into MHANPs under different loading conditions. The synthesized MHANPs and IBU-MHA particles were confirmed by X-ray diffraction (XRD), fourier-transform infrared spectroscopy (FTIR), brunauer–emmett–teller (BET), transmission electron microscopy (TEM), and thermal analysis (TGA). Drug loading (DL) efficiency of IBU-MHA particles was determined by ultraviolet–visible (UV-Vis) spectroscopy, and indicated that the optimized IBU-MHA particles with high DL (34.5%) can be obtained at an IBU/ MHANPs ratio of 35/50 (mg/mg), impregnation period of 24 h, and temperature of 40 °C using ethanol as solvent. *In-vitro* drug release test was carried out to prove the efficiency of IBU-MHA particles as a sustained drug delivery system. A more sustained and controlled drug release was observed for this particles, indicating that it may be have good potential as drug reservoirs for local drug release.

## Introduction

In recent years, there has been an increased interest in the development of controlled release drug delivery systems, which is an efficient technique for the biomedical applications. Such drug delivery systems show many advantages as compared to conventional forms, such as increased bioavailability, more effectiveness and safety, controlled and sustained release profile and predictable therapeutic response. In general, an ideal and effective delivery system should be safe, biocompatible, easy to fabricate, mechanically strong, simple to apply, and able to transport the desired amount of therapeutic bioactive molecules such as drug, protein, and peptide to the targeted organs and release the drug in a controlled manner (1-3). 

So far, a large number of systems have been applied for controlled and sustained release of bioactive molecules, including Layered double hydroxides (LDH) nanoparticles nanocomposite polymers, mesoporous hydroxyapatite nanoparticles, polymeric hydrogels, and mesoporous silica nanoparticles (4-13). Among the many different systems that have been developed for controlled and sustained release applications, mesoporous hydroxyapatite nanoparticles (MHANPs) have attracted particular attention owing to their many desirable properties (14, 15).

Hydroxyapatite (HA), Ca_10_(PO_4_)6(OH)_2_, is a calcium phosphate similar to the human hard tissues such as bones and teeth in morphology and composition, which make it have great applications in tissue engineering in the dental and orthopedic fields (16, 17). It possesses a hexagonal structure and a stoichiometric Ca/P ratio of 1.67. As compared to other calcium phosphates, HA has high stability under physiological conditions as temperature, pH and composition of the body fluids. HA with various morphologies and surface properties is extensively used for controlled release of biomolecules such as drug, protein and peptide due to its excellent biocompatibility, bioactivity, nontoxicity and non-inflammatory nature (18). In addition, MHANPs have attracted great attention in fields of chemistry, physics, electronics, optics, and materials science (19). Recently, ordered mesoporous hydroxyapatite nanoparticles (MHANPs) have been synthesized via different synthesis techniques such as self-assembly, spray drying, double emulsion, solvo-thermal, sol-gel and hydrothermal, which can be proposed as potential delivery systems due to their excellent properties such as high surface area, tunable pore size, and high pore volume (11, 20). Such unique properties are effective for their high drug loading efficiency and controlled drug release. Porous HA particles have a great potential as drug carrier due to the following advantages: (i) they have excellent biocompatibility, and suitable physicochemical properties such as a large surface area and high pore volume, which make it possible to load a high amount of drug molecules and release at a sustained and controlled manner (21), and (ii) the OH groups in HA particles can adsorb the drug molecules by the hydrogen bonding interactions, resulting in improving the drug loading capacity and release profile (14). Thus, MHANPs impregnated with the various drugs form a MHA-drug particles that results in the controlled release of drugs into the targeted site (22, 23). Researchers have used MHANPs to deliver various bioactive molecules, such as genes, peptides and proteins, and drugs. For example, Ya-Ping Guo *et al.* prepared Mesoporous carbonated hydroxyapatite microspheres via hydrothermal technique for controlled release of vancomycin. Under *in-vitro* conditions, these microspheres exhibited a controlled release profile for the incorporated drug (24). 

However, there are no published data on the various parameters (drug/MHANPs ratio, impregnation period, reaction temperature, and solvent type) affecting the drug loading efficiency of drug-MHA particles. Since, the fabrication of drug-MHA particles with high drug loading capacity has high importance in the pharmaceutical sciences, investigation of effective parameters on the drug loading efficiency of drug-MHA particles is necessary.

Ibuprofen (IBU) is one of the most common analgesic anti-inflammatory nonsteroidal drugs (NSAIDs) used for the relief of pain, fever, and inflammation. This includes migraines, rheumatoid arthritis, and so on (25). It is believed that the action mechanism of IBU is based on inhibiting two isoforms of cyclooxygenase; COX-1 and COX-2 enzymes involved in the synthesis of prostaglandins (PG). Analgesic, antipyretic, and anti-inflammatory activity of NSAIDs appeared to be due to blocking the synthesis of PG (26). On the other hand, it has been reported that IBU could also cause adverse effects such as nausea, dyspepsia, diarrhea, constipation, gastrointestinal ulceration/bleeding, headache, dizziness, rash, salt and fluid retention, and hypertension (27). We suggest that these adverse effects can be reduced by incorporation of IBU into MHANPs, because they release drug in a controlled manner, so a desirable amount of drug can be delivered to targeted site. IBU has been loaded into various carriers for sustained release such as polymeric carriers, inorganic particles and polymeric–inorganic hybrid materials (28-30). The carrier surface area is a substantial parameter in controlling the drug absorption and release rate (31). Among the ibuprofen-loaded carriers studied, few show both high loading capacity and ideal sustained release properties in release media. 

Based on the above background, fabrication of IBU-MHA particles as a drug delivery system and investigation of effective parameters on its drug loading efficiency are quite attractive and—to the best of our knowledge—there is still no related report. In this study, MHANPs were synthesized by a self-assembly process. Then, IBU-MHA particles was fabricated via the incorporation of IBU into MHANPs using an impregnation process. The optimization of the incorporation of IBU molecules into MHANPs was carried out under different loading conditions, which is the main goal of the present study. 

## Experimental

*Materials*

Cetyltrimethylammonium bromide (CTAB, Merck, German), sodium hydroxide (NaOH, Merck, German), hydrochloric acid (HCl, Merck, German), potassium chloride (KCl, Merck, German), sodium dihydrogen phosphate (NaH_2_PO_4_, Merck, German), 1-dodecanethiol (C12-SH, Merck, German), ethanol (C_2_H_5_OH, Merck, German), calcium nitrate (Ca (NO_3_)_2_·4H_2_O, Merck, German), ortho phosphoric acid (85% purity, Merck, German), ammonia solution (25% extra pure, Merck, German), Ibuprofen powder (IBU, Hakim Pharmaceutical Co., Tehran, Iran) were used as received without further purification. Double distilled water was used throughout the present study.

*Synthesis of MHANPs*

MHANPs were synthesized according to a self-assembly technique described by Bakhtiari *et al*. (32). First, an emulsion containing CTAB (10 g), C12-SH (10 g) in 1 L of double distilled water was prepared and with the addition of 50 mL NH₃ (25%) the pH of reaction mixture was adjusted at pH 12.5 using ammonia solution. The glass beaker contents magnetically stirred at 60 °C for 30 min. Hydroxyapatite precursors (7.8 g di-Ammonium hydrogen phosphate (DAP) and 23.5 g Calcium nitrate tetrahydrate) were separately dissolved in 100 mL of double distilled water. Then they added to above solution slowly within 15 min and stirred for 2 h in 60 °C. After that, the final mixture was passed through a cotton filter and then the white precipitate was filtered through a paper filter and washed three times with enough amount of double distilled water to eliminate the unreacted components and impurities, and dried at 120 °C for 6 h. The resultant white precipitate was ground using a mortar and pestle. The final white powder was calcined at 550 °C for 6 h with a heating rate of 1 °C/min.

*Fabrication of MHA-IBU particles*

MHA-IBU particles were fabricated by reacting MHANPs suspension with an alcoholic solution containing the dissolved IBU using an impregnation process. Hence, 100 mg MHANPs was dispersed in 25 mL solvent for 4 h at the room temperature. Separately, various amounts of IBU was dissolved in 25 mL solvent and added into the MHANPs suspension with constant stirring at the desired temperature. The suspension was then incubated at the desired temperature with shaking for sufficient duration to allow IBU incorporation into the pores of MHANPs to equilibrate. Then, the final mixture was separated by centrifugation at 2000 rpm, washed three times with enough amount of solvent to eliminate the unreacted IBU, and dried at 50 °C under vacuum for 24 h. The dried MHA-IBU particles were ground using a mortar and pestle, sieved through a 180-μm sieve and maintained in a desiccator. The experiments were performed to optimize the MHANPs: IBU weight ratio (5:1 to 1:1), temperature (30 to 80 °C), ethanol/water ratio (100:0 to 40:60%), and time (10 to 60 h) required for maximum incorporation of IBU into the pores of MHANPs. The concentration of IBU in filtrate was measured using UV-Vis spectroscopy at λmax = 222 nm.

*Characterization*

*X-ray Diffraction*

Crystallographic structure of the synthesized MHANPs and MHA-IBU particles was evaluated via powder X-ray Diffraction (PXRD) (STOE-STADI, Germany) at room temperature. The analyses were performed using a STOE-STADI powder diffractometer system with Cu-Kα (λ = 1.54060 °A) at 40 keV and 40 mA and step length of 0.06 º with step time of 1 sec in the scan range of 2θ from 2 to 80 °.

*Fourier Transform Infrared Spectroscopy*

The chemical composition of the synthesized MHANPs and MHA-IBU particles was investigated using a FTIR spectrophotometer (Thermo Nicolet NEXUS 670 FTIR) in the wavenumber range of 4000–400 cm^−1^ at a resolution of 0.5 cm^-1^. Test samples were mixed with KBr powder and pressed into a suitable disk in order to measure their FTIR spectra. 

*Scanning Electron Microscope*

The morphological properties of the synthesized MHANPs and MHA-IBU particles were studied via scanning electron microscope (SEM) (KYKY-SEM 3200). Test samples were first coated with a thin gold layer before microscopy and their SEM images were collected at an accelerating voltage of 26 kV with different magnifications.

*Transmission Electron Microscope*

The size and shape of the synthesized MHANPs were investigated via transmission electron microscopy (TEM). MHANPs powder was dispersed in ethanol under ultrasonication with an ultrasonic probe for 30 min and a drop of the resultant suspension was deposited on carbon-coated copper grids. TEM images were collected digitally using a ZIESS EM900 TEM at an accelerating voltage of 80 kV.

*Thermal analysis (TGA)*

TGA-50H thermogravimetric analyzer (TGA-50H, SHIMADZU) was used to investigate the thermal stability and degradation of the synthesized MHANPs and MHA-IBU particles. For TGA analysis, weighted amount of test samples were sealed in an alumina pan and then heated from room temperature to 700ºC at a heating rate of 10ºC min^-1^ under a constant nitrogen flow rate (20 mL min^-1^).

*Nitrogen adsorption analysis*

Data were collected using a Quantachrome Autosorb-1 gas adsorption analyzer at 77 K after degassing the samples at 473 K for 24 h. The specific surface area and pore size distribution of the synthesized MHANPs and MHA-IBU particles powders were calculated according to the Brunauer-Emmett-Teller (BET) and Brunauer-Joyner-Halenda (BJH) techniques (BELSORP, Japan), respectively.

*Drug loading efficiency*

To measure content of IBU incorporated into the MHA-IBU particles, the final suspension containing MHA-IBU particles and free IBU was centrifuged at 2000 rpm for 10 min, and supernatants of the suspension were collected. Then, collected supernatants were diluted with suitable solvent (ethanol/water mixture), and filtered via a cellulose acetate membrane (0.45-µm). The content of IBU presented in the supernatants was measured using an UV–Vis spectrophotometer technique at λmax = 222 nm. The content of IBU incorporated into the MHA-IBU particles was calculated as the difference between the initial content of IBU and the content of IBU presented in the supernatants. The drug loading (DL%) of the MHA-IBU particles was determined according to Equation 1.

(1) Drug Loading DL%=weight of IBU incorporated (mg)weight of MHA-IBU nanohybrid (mg)×100

*Drug release analysis*

The *in-vitro* drug release profile of test samples was performed in the buffer phosphate solution (PBS, pH 4.5 and 7.4). Suitable amounts of IBU, MHA-IBU particles were immersed in 100 mL of buffer at 37 ± 0.5 °C and stirred at 50 rpm. At predetermined time intervals, 5 mL aliquots of the buffer solution were withdrawn from the release medium and the same volume of fresh buffer phosphate solution were added into the release medium. The aliquots—after suitable dilution and filtration—were analyzed using UV-vis spectroscopy (UV-1280, SHIMADZU) at 222 nm, and the content of IBU was calculated via a standard calibration curve obtained under the same conditions. These analyses were repeated three times, and the results were reported as mean values.

*Drug release kinetics study*

In order to predict and correlate the release profile of IBU of MHA-IBU particles in release medium (pH 7.4), the IBU release kinetics of the MHA-IBU particles were studied using different kinetic models:

Zero order model                      $f_{t}=k_{0}t$

First order model                     $\log\left(1-f_{t}\right)=\frac{k_{1}t}{2.303}$

Higuchi model                     ft=kHt12

Korsmeyer–Peppas model                      $f_{t}=k_{P}t^{n}$

Where f_t_ is the fraction of drug released at time t, t is the release time, k_0_, k_1_, k_H_ and k_P_ shows the zero-order release, first-order release, Higuchi dissolution, and rate constant, respectively and n is the release exponent, showing the type of the drug release mechanism. According to the korsmeyer–peppas equation, amounts of n between 0.43 and 0.85, n ≥ 0.85 and n ≤ 0.43 are indicative the both diffusion and swelling controlled drug releases, relaxation-controlled release and diffusion-controlled release, respectively (33).

## Results and Discussion

*FTIR analysis*

**Figure 1:**
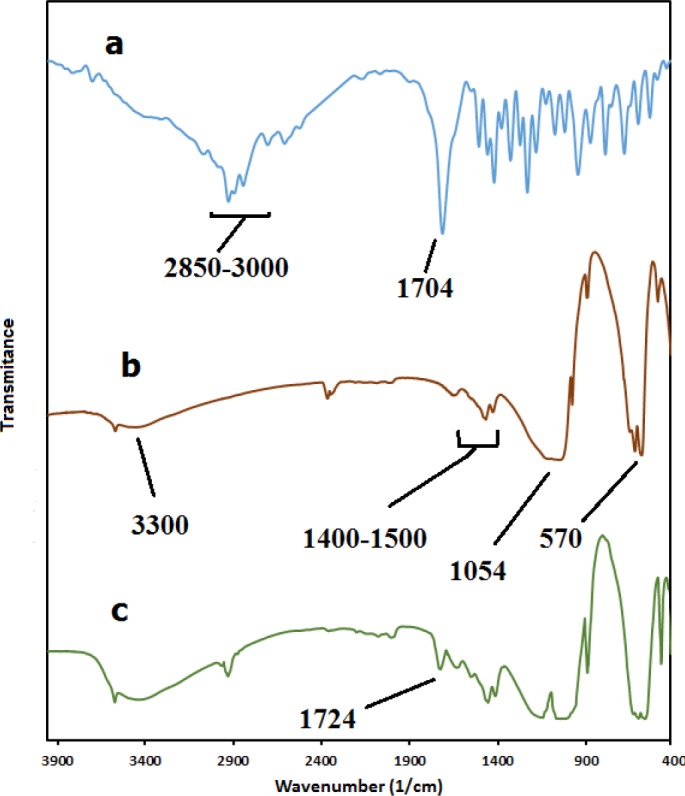
FTIR spectra of (a) ibuprofen (IBU), (b) MHANPs, (c) MHA-IBU particles

FTIR analysis of the pure IBU, MHANPs and MHA-IBU particles was carried out to investigate their chemical structure and to identify the functional groups presented in test samples, and the results are shown in Figure 1. As is obvious in Figure 1a, the FTIR characteristic peaks of IBU were observed in the wavenumber range of 2850 – 3000 cm^-1^ (C–H stretching vibration), 1704 cm^-1^ (COOH asymmetrical stretching vibration), and 1511 and 1419 cm^-1 ^(C–C stretching vibration in aromatic ring). In the FTIR spectrum of the synthesized MHANPs (Figure 1b), the characteristic absorption band at around 3300 cm^−1^ is due to the stretching vibrational mode of OH functional group. The absorption bands at 570 and 1054 cm^−1^ are attributed to the vibrational modes of anions. The absorption bands related to the vibrational modes of are observed in the range of 1400–1500 cm^−1^. These bands appeared in the FTIR spectrum confirmed the successful synthesis of MHANPs via self-assembly method. Also, the FT-IR technique was applied to confirm the formation of MHA-IBU particles. As shown in Figure 1c, the FTIR spectrum of the MHA-IBU particles represents two types of bands: One was related to the IBU molecules and the other was caused by the MHANPs. In addition, comparing these spectra clearly shows that absorption band related to the COOH asymmetrical stretching vibration shifts the higher wavenumbers from 1704 cm^−1^ to 1724 cm^−1^. Similar shifts have been reported in the literature. This behavior may be due to the hydrogen bonding interactions between the OH group of MHANPs and the COOH group of IBU molecules, indicating the successful incorporation of IBU molecules into the pores of MHANPs.

*XRD analysis*

**Figure 2:**
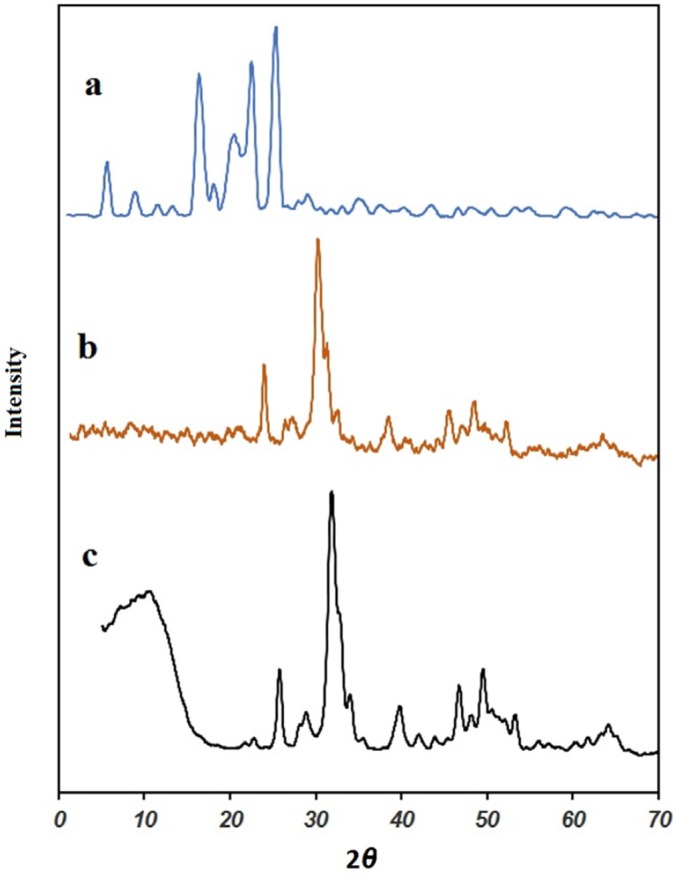
XRD pattern of (a) ibuprofen (IBU), (b) MHANPs, (c) MHA-IBU particles

In Figure 2, the XRD patterns of the pure IBU, MHANPs and MHA-IBU particles synthesized under optimized conditions are displayed in the 2θ range of 10–80 º. The XRD pattern of the synthesized MHANPs exhibits a characteristic diffraction peaks at 2θ = 26 and 32 °, which corresponds to the (0 0 2) and (1 1 2) (h k l) planes (JCPDS File No. 09-0432), indicating the formation typical phases of MHANPs (34, 35). According to the phase analysis, MHANPs synthesized by this method has high purity, and no impurity phase was detected in the pattern. Also, the strong diffraction peaks indicated that the synthesized MHANPs were well crystallized. As is obvious in Figure 2c, the XRD pattern of the MHA-IBU particles does not show characteristic diffraction peaks related to the pure crystalline IBU powder, and exhibits only characteristic reflections of a crystalline MHANPs. The most probable explanation of the absence of characteristic diffraction peaks of IBU in the XRD pattern of the MHA-IBU particles is the presence of a well dispersed thin layer of IBU molecules into pores of MHANPs via hydrogen bonding interactions between OH and COOH groups MHANPs and IBU.

*SEM, TEM and DLS analysis*

**Figure 3:**
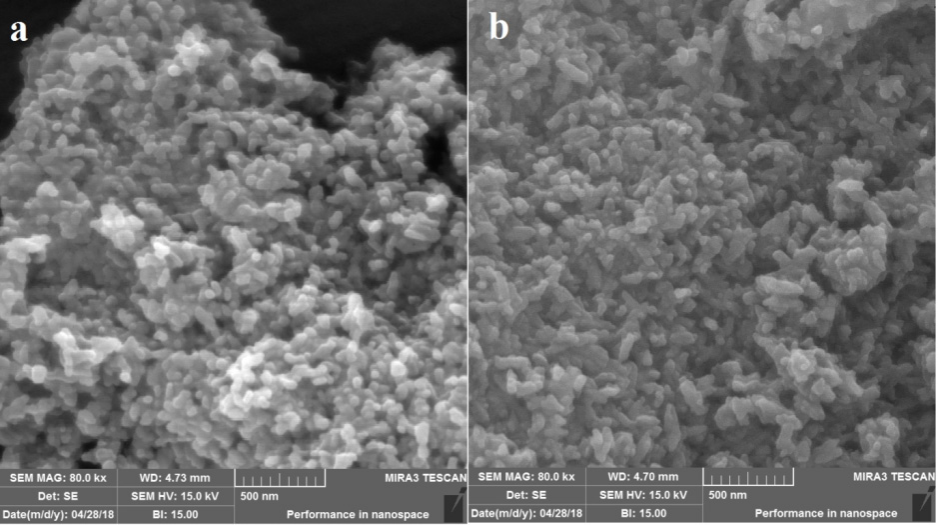
The SEM images of (a) MHANPs, (b) MHA-IBU particles

**Figure 4:**
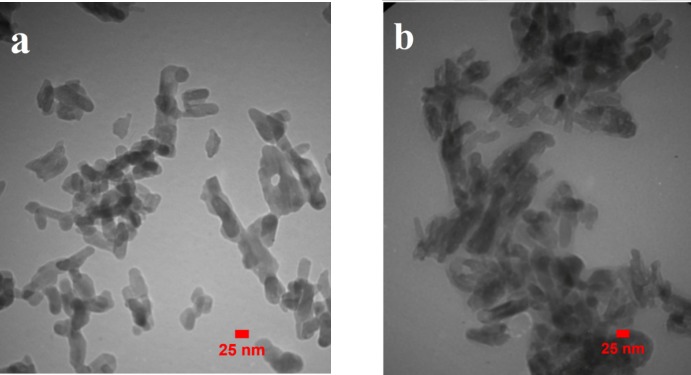
The TEM images of (a) MHANPs, (b) MHA-IBU particles

**Figure 5:**
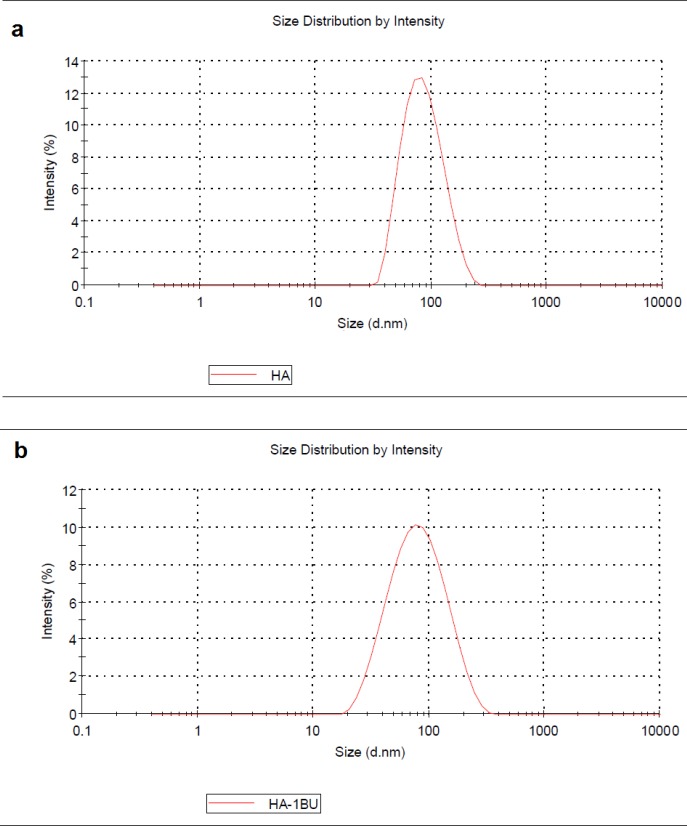
The size distribution of (a) MHANPs and (b) MHA-IBU particles

The external morphological properties of MHANPs and MHA-IBU particles synthesized under optimized conditions were studied by SEM technique. As indicated in Figure 3, the synthesized MHANPs exhibit irregular morphology, which can be attributed to non-uniform crystal growth in crystallographic directions. Also, the morphology of MHA-IBU particles is quite similar to that of the MHANPs. This may be due to the incorporation of IBU molecules into pores of MHANPs, resulting in no effect on the crystal growth during MHA-IBU particles formation. It can be said that the different particle sizes and surface areas contribute to the distribution state of the synthesized MHANPs and MHA-IBU particles. Therefore, the size, shape and porous structure of the synthesized MHANPs and MHA-IBU particles are further investigated using TEM and DLS, and the results are shown in Figures 4 and 5. Both MHANPs (Figure 4a) and MHA-IBU particles (Figure 4b) particles are irregular crystals of about 13 nm × 67 nm (MHANPs) and about 19 nm × 78 nm, respectively. It is confirmed using DLS technique that the average diameter of MHANPs and MHA-IBU particles are 73.4 ± 24.5 and 83.9 ± 32.1 nm, respectively. The larger diameter of MHA-IBU particles as compared to the MHANPs can be due to the agglomeration of MHA-IBU particles during fabrication process. Polydispersity index (PDI) of MHANPs and MHA-IBU particles are 0.28 and 0.39, respectively. This indicates that the fabricated particles have a good size distribution.

*BET analysis*

The porous structure of mesoporous materials as a drug delivery system is one of the most important factors determining the drug loading efficiency and drug release profile. In other words, the pore size, surface area and volume of mesoporous materials have great influence on the drug loading efficiency and drug release profile. Thus, the pore size, surface area and volume of the synthesized MHANPs and MHA-IBU particles were also investigated using the Brunauer-Emmett-Teller (BET) and Brunauer-Joyner-Halenda (BJH) techniques.

**Figure 6:**
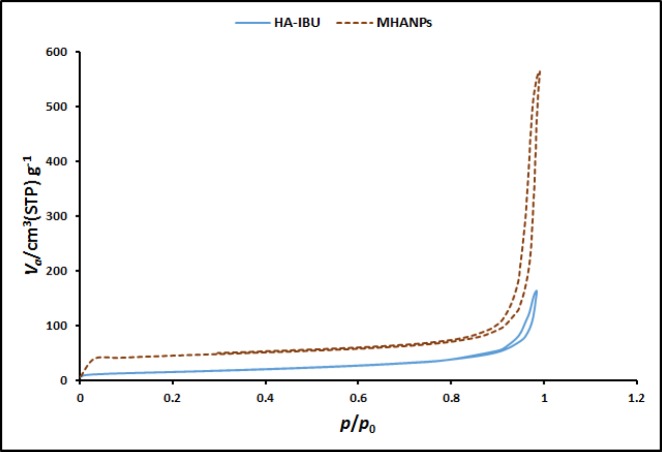
The nitrogen adsorption–desorption isotherms of the synthesized MHANPs and MHA-IBU particles

The nitrogen adsorption–desorption isotherms of the synthesized MHANPs and MHA-IBU particles are shown in Figure 6. It is obvious that nitrogen isotherms of test particles are different. The hysteresis loop for MHANPs are wide, with larger pore size as compared to MHA-IBU particles, which have narrow loops and smaller pore size. This behavior can be due to existence of high ionization degree of 1-dodecanethiol and more RS^−^ ions, resulting in swelling the micelle and increasing in the pore size, where as in the MHA-IBU particles, the incorporation of IBU molecules into the pores of MHANPs leaded to decreasing the pore size.

Also, the physical characteristics (the pore size, surface area and volume) of test particles are summarized in Table 1, indicating the same trend as the pore size for the pore volume (V_P_) and BET surface area. In other words, the pore size, pore volume and surface area of MHA-IBU particles were significantly reduced as compared to MHANPs. These results confirmed that the formation of mesoporous MHANPs was successful, and IBU molecules were successfully loaded into the pores of MHANPs.

*TGA analysis*

**Figure 7:**
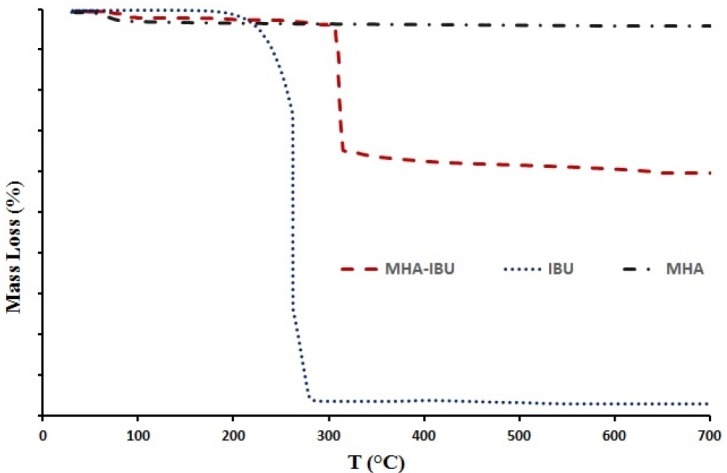
The thermal behavior of the pure IBU, MHANPs and MHA-IBU particles particles

The thermal behavior of the pure IBU, MHANPs and MHA-IBU particles synthesized under optimized conditions was investigated via TGA technique. As shown in Figure 7, dried and pure IBU powder presented a mass loss of about 96.5 wt% (between 200 and 280 °C) (36). The synthesized MHANPs showed a minor weight loss of about 4.5 wt% at temperature below 200 °C, which could be due to the evaporation of the trapped and adsorbed water molecules (37). Also, a main weight loss pattern appeared in the temperature range of 285–360 ºC (about 35.6 wt%) for MHA-IBU particles, which may be related to the evaporation of IBU molecules incorporated into the pores of MHANPs. Thus, the drug loading of MHA-IBU particles could be calculated to be approximately 35.6 wt%. It is clear that the evaporation temperature of IBU molecules in the MHA-IBU particles shifts to higher temperature compared to the pure IBU molecules, indicating the incorporation of IBU molecules into the pores of MHANPs. This phenomenon can be described based on the restriction of molecular motion of IBU molecules in the pores in combination with the hydrogen bonding interactions between OH group on the pore wall and COOH group of the IBU molecules. Effectively, this resulted in a lower vapor pressure of IBU molecules incorporated into the pores of MHANPs and, hence, to a higher evaporation temperature. These results indicate that the MHA-IBU particles have a high drug loading capacity, and could be applied as a drug delivery system.

*Drug loading efficiency analysis*

*Time effect*

**Figure 8:**
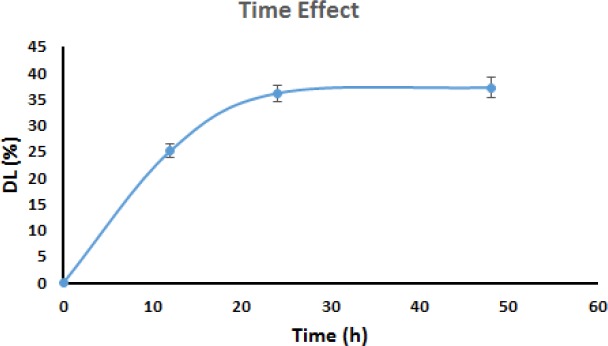
The relationship between IBU incorporation and time

Figure 8 exhibits the amount of IBU incorporated into the pores of MHANPs in the time range of 12–48 h. As indicated in Figure 8, the amount of IBU incorporated into the pores of MHANPs increased with increasing loading time, until incorporation equilibrium was established within 24 h (*P* < 0.05 for t12–t24 and *P* > 0.05 for t24–t48). For example, the incorporation of IBU into the pores of MHANPs reached a constant value of about 34.2% after 24 h of soaking. 

This phenomenon can be explained by two factors: 1) time for the dissolution of IBU in the solvent and 2) time required for the diffusion of the dissolved IBU into the pores of MHANPs. Since the IBU powder is rapidly dissolved in the solvent (ethanol), it can be said that IBU diffusion, and not IBU dissolution, is the rate-limiting step for incorporation of IBU into the pores of MHANPs. Thus, the loading time was set to 24 h in the subsequent experiments to avoid the partial incorporation of IBU molecules into the pores of MHANPs.

*Temperature effect*

**Figure 9:**
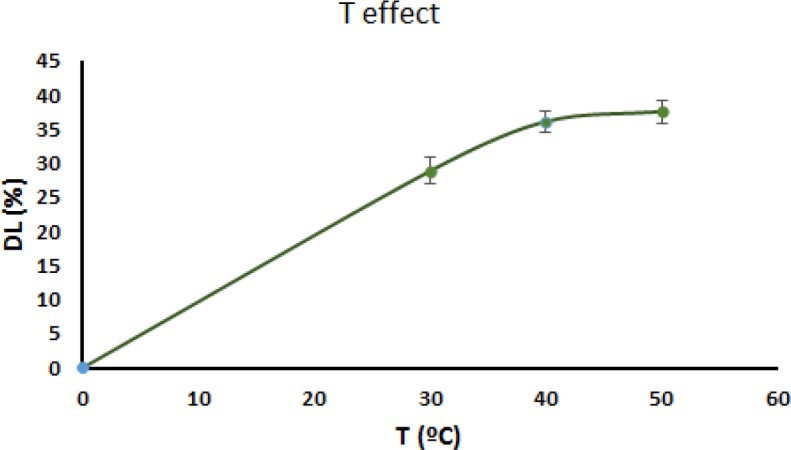
The relationship between IBU incorporation and temperature

The relationship between IBU incorporation and loading temperature is exhibited in Figure 9. As is indicated in this figure, the content of IBU incorporated into MHANPs increased by increasing the temperature up to 40 °C, and after that, it remained constant (*P* < 0.05 for T30–T40 and *P* > 0.05 for T40–T50). Since, the concentration gradient at a certain point along the diffusion path depends on interaction time, diffusion condition is described using Fick’s second law (1), which is a second-order differential Equation (38).

(1) $$\left(\frac{\partial C_{x}}{\partial t}\right)=D\frac{\partial^{2}C_{x}}{\partial X^{2}}$$

Where D, the diffusion coefficient, can be expressed according to Equation 2.

 (2) D=D0e-qkT

According to Equation 2, D, the diffusion coefficient, depends on the temperature, which the higher temperature results in promoting more diffusion processes. Since there were no significant differences in the content of IBU incorporated into MHANPs at temperatures from 40 °C to 50 °C, 40 °C was chosen as suitable temperature because of the risk of IBU degradation at higher temperatures.

*Solvent type effect*

**Figure 10:**
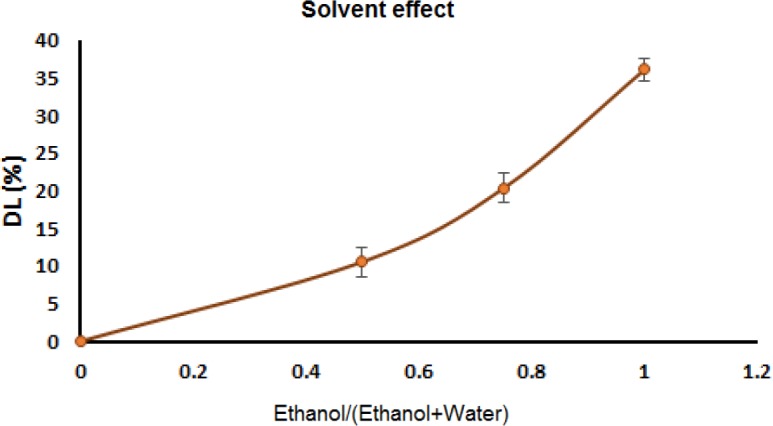
The ethanol/(ethanol+water ratio) effect on IBU incorporation efficiency

The solubility of drug molecules in the solvent can play a crucial role in the drug incorporation into the pores of mesoporous materials. Thus, the effect of the solvent type on the incorporation of IBU molecules into the pores of MHANPs was investigated in the different ethanol/(ethanol+water) ratios of .05, 0.75 and 1.0, and the results are shown in Figure 10. As indicated in this figure, the drug loading efficiency of the MHA-IBU particles increased by increasing the content of ethanol in the water/ethanol mixture used for IBU dissolution (*P* < 0.05). A possible explanation for this phenomenon might be that the major mechanism of IBU incorporation into MHANPs is based on an adsorption process, which is occurred via the formation of hydrogen bonding interactions between carboxyl group of IBU molecules and hydroxyl groups in the MHANPs. Thus, there is a competition between IBU molecules and solvents that have the ability to form hydrogen bonds. Since the hydrogen bonding ability of water with hydroxyl groups of MHANPs is greater than that of ethanol, water molecules via the formation of hydrogen bonding with hydroxyl groups of MHANPs will significantly hinder the adsorption of IBU molecules into pores of MHANPs. Thus, the drug loading efficiency of the MHA-IBU particles reduced remarkably by increasing the content of water in the solvents mixtures. Based on these results, ethanol was chosen as a suitable solvent for the IBU dissolution.

**Figure 11:**
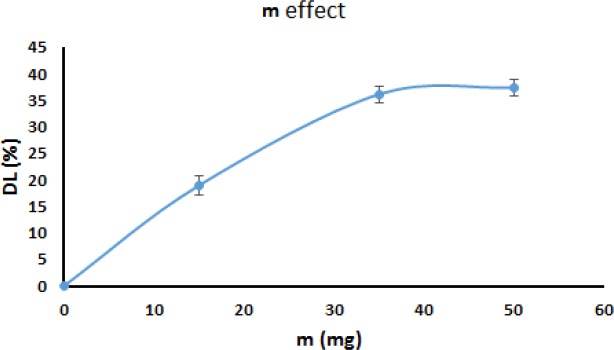
IBU initial amount effect on IBU incorporation efficiency

**Figure 12:**
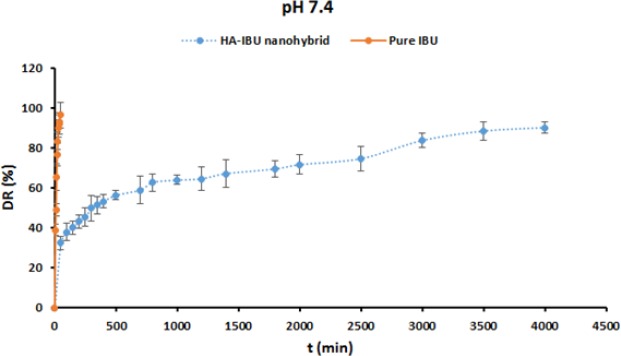
*In-vitro *drug release behavior of the pure IBU, and MHA-IBU particles at pH values of 4.5 and 7.4. Each point is the mean ± SD, n = 3

**Table 1:** The physical characteristics (the volume, surface area and pore size) of test particles

**Samples**	**S** **BET ** **(m /g)** **2**	**Vp (cm** **3** **/g)**	**BJH (nm)**
MHANPs	60.38	0.803	39.01
MHA-IBU	54.7	0.252	18.31

**Table 2:** Kinetic model parameters for MHA-IBU particles; n: kinetic exponent; R2: regression coefficient

**Variables**	**MHA-IBU particles**
N	0.28
$R_{0}^{2}$	0.74
$R_{1}^{2}$	0.92
$R_{H}^{2}$	0.97

*IBU initial amount effect*

As is obvious in Figure 11, the drug loading efficiency of the MHA-IBU particles is affected by the initial IBU amount. According to this figure, the drug loading efficiency of the MHA-IBU particles increased by increasing the initial amount of IBU in the solution, until the solution was saturated with IBU molecules (*P *< 0.05 for m1–m2 and *P* > 0.05 for m2–m3). A main reason for this phenomenon can be that in saturated solution, more IBU molecules are expose to MHANPs, which results in the incorporation of more IBU molecules into MHANPs. Saturated solution was obtained by dissolving 35 mg IBU in 10 mL ethanol*.* Thus, 35 mg IBU was considered as optimized amount of IBU used for the fabrication of drug-MHA particles.

*Drug release analysis*

The *in-vitro* release behavior of IBU from test samples was studied in the phosphate buffer solution (PBS, pH 7.4). Two types of formulations—including pure IBU, and MHA-IBU particles particles—were applied for this purpose. The results are shown in Figure 12. The pure IBU powder in the release medium shows a rapid burst release of IBU in short time period. A possible explanation for this might be that COOH groups of IBU molecules could disassociate and change from the COOH group to COOˉ group at pH 7.4, resulting in high solubility in buffer phosphate. Also, ionic strength could improve the solubility of IBU in phosphate buffer (pH 7.4). High solubility of IBU powder in phosphate buffer resulted in the burst release of pure powder in the release medium (39, 40).

Also, as is obvious in Figure 12, there is a significant difference between release profile of IBU from the pure IBU powder, and MHA-IBU particles in both release media. MHA-IBU particles exhibited an initial burst drug release for 100 min, followed by a relatively slow release until 4500 min. Initial burst release of IBU from the MHA-IBU particles could be due to the adsorption of IBU molecules on the surface of MHANPs. After the initial burst release, the amounts of IBU released from the MHA-IBU particles in the release medium were maintained at approximately 37.8 wt% under pH 7.4. The high content of IBU released from the MHA-IBU particles at pH 7.4 can be described via the following reasons. First, COOH group on the structure of IBU incorporated into pores of MHANPs could disassociate and change from the COOH group to COO^ˉ^ group at pH 7.4. Thus, the hydrogen bonding interactions between IBU and MHANPs cannot form. Second, IBU due to the COOH disassociation has high solubility. Thus, the release rate of IBU increases by increasing pH value of the release medium. In addition, it was found that the amount of IBU released from the MHA-IBU particles in the release medium had a limiting value. The IBU released from device did not enhance even at prolonged time interval. This behavior may be due to this phenomenon that porosity of the HA mesoporous particles significantly enhances the storage time of the IBU into their pores and has a remarkable effect on the release rate. In this study, after 60 h, the release rate has reached about 90%, a common behavior in drug delivery systems with controlled release (14, 41). Gu *et al.* synthesized doxorubicin (DOX)-HA particles and investigated their release profile. They found that the released DOX amounts in release medium had a limiting value, and amount of DOX did not increase even at prolonged withdrawal time interval, indicating that the DOX-loaded HA had a slow, long-term, and steady release rate (42). This phenomenon showed that the MHA-IBU particles had a slow, prolonged, and steady release rate, leading to inhibiting the explosive release of IBU from them and prolonging their therapeutic effect.

*Drug release kinetics*

The release rate of IBU from the MHA-IBU particles was studied based on Korsmeyer-Peppas kinetic model in the release medium (pH 7.4). The obtained results are summarized in Table 2. As is known in Table 2, For MHA-IBU particles, where n value is lower than 0.43, the drug release was controlled based on Fickian transport, most probably due to the increased resistance to swell and erode in the release medium.

Also, in order to investigate the release behavior of IBU from the MHA-IBU particles in the release medium, the drug release data obtained from these particles were fitted to various kinetic models including the zero-order, first-order and Higuchi models as listed in Table 2. 

The correlation coefficient () for the Higuchi model is much higher than and for the zero-order and first-order models. This means the release kinetics of IBU from the MHA-IBU particles follows the Higuchi model. The release constant of the Higuchi model (k_H_) for MHA-IBU particles at pH 7.4 is equal to 1.2.

## Conclusion

We successfully synthesized MHANPs and MHA-IBU particles a novel sustained-release drug delivery system by self-assembly and impregnation processes, respectively. We have characterized in detail the structural, thermal properties of MHANPs and MHA-IBU particles using XRD, FT-IR, BET, TEM, SEM, DLS and TGA technique. Also, we have shown that MHA-IBU particles release IBU molecules in a sustained and controlled manner. As a result, the MHA-IBU particles could be an idea candidate for biomedical and pharmaceutical applications, such as sustained and controlled release drug delivery systems.
